# DNA demethylation triggers cell free DNA release in colorectal cancer cells

**DOI:** 10.1186/s13073-024-01386-5

**Published:** 2024-10-09

**Authors:** Valeria Pessei, Marco Macagno, Elisa Mariella, Noemi Congiusta, Vittorio Battaglieri, Paolo Battuello, Marco Viviani, Giulia Gionfriddo, Simona Lamba, Annalisa Lorenzato, Daniele Oddo, Fariha Idrees, Alessandro Cavaliere, Alice Bartolini, Simonetta Guarrera, Michael Linnebacher, Laura Monteonofrio, Luca Cardone, Michele Milella, Andrea Bertotti, Silvia Soddu, Elena Grassi, Giovanni Crisafulli, Alberto Bardelli, Ludovic Barault, Federica Di Nicolantonio

**Affiliations:** 1 Candiolo Cancer Institute, FPO-IRCCS, Candiolo, Turin, Italy; 2 Department of Oncology, University of Torino, Turin, Italy; 3 IFOM, the AIRC Institute of Molecular Oncology, Milan, Italy; 4 IIGM-Italian Institute for Genomic Medicine, c/o IRCCS, Candiolo, Turin, Italy; 5 Clinic of General Surgery, Molecular Oncology and Immunotherapy, UMR, Rostock, Germany; 6 Department of Research and Advanced Technologies, Regina Elena National Cancer Institute IRCCS, Rome, Italy; 7 Section of Innovation Biomedicine - Oncology Area, Department of Engineering for Innovation Medicine, University of Verona and Verona University and Hospital Trust, Verona, Italy

**Keywords:** Colorectal cancer, cfDNA, Cell cycle, Cell death, MSI, DNA methylation, Liquid biopsy

## Abstract

**Background:**

Liquid biopsy based on cell-free DNA (cfDNA) analysis holds significant promise as a minimally invasive approach for the diagnosis, genotyping, and monitoring of solid malignancies. Human tumors release cfDNA in the bloodstream through a combination of events, including cell death, active and passive release. However, the precise mechanisms leading to cfDNA shedding remain to be characterized. Addressing this question in patients is confounded by several factors, such as tumor burden extent, anatomical and vasculature barriers, and release of nucleic acids from normal cells. In this work, we exploited cancer models to dissect basic mechanisms of DNA release.

**Methods:**

We measured cell loss ratio, doubling time, and cfDNA release in the supernatant of a colorectal cancer (CRC) cell line collection (*N* = 76) representative of the molecular subtypes previously identified in cancer patients. Association analyses between quantitative parameters of cfDNA release, cell proliferation, and molecular features were evaluated. Functional experiments were performed to test the impact of modulating DNA methylation on cfDNA release.

**Results:**

Higher levels of supernatant cfDNA were significantly associated with slower cell cycling and increased cell death. In addition, a higher cfDNA shedding was found in non-CpG Island Methylator Phenotype (CIMP) models. These results indicate a positive correlation between lower methylation and increased cfDNA levels. To explore this further, we exploited methylation microarrays to identify a subset of probes significantly associated with cfDNA shedding and derive a methylation signature capable of discriminating high from low cfDNA releasers. We applied this signature to an independent set of 176 CRC cell lines and patient derived organoids to select 14 models predicted to be low or high releasers. The methylation profile successfully predicted the amount of cfDNA released in the supernatant. At the functional level, genetic ablation of DNA methyl-transferases increased chromatin accessibility and DNA fragmentation, leading to increased cfDNA release in isogenic CRC cell lines. Furthermore, in vitro treatment of five low releaser CRC cells with a demethylating agent was able to induce a significant increase in cfDNA shedding.

**Conclusions:**

Methylation status of cancer cell lines contributes to the variability of cfDNA shedding in vitro. Changes in methylation pattern are associated with cfDNA release levels and might be exploited to increase sensitivity of liquid biopsy assays.

**Supplementary Information:**

The online version contains supplementary material available at 10.1186/s13073-024-01386-5.

## Background

Precision oncology relies on the early diagnosis of cancer, detection of minimal residual disease, and timely characterization of the tumor molecular landscape to identify actionable alterations that can guide patient treatment [1–4]. Blood from most cancer patients contains cell-free DNA (cfDNA) released predominantly by neoplastic cells, although the contribution of normal tissue can be substantial under certain circumstances [5–7]. Tumor-derived cfDNA has emerged as a minimally invasive biomarker to deliver precision oncology [8]. Despite generally increased levels of cfDNA in cancer patients compared to healthy individuals, its amount is often insufficient to intercept cancer or detect the presence of residual disease after surgery [9, 10]. To overcome the limited sensitivity of total cfDNA assessment, several liquid biopsy assays encompass the determination of tumor specific molecular alterations in cfDNA, including somatic sequence variants, methylation patterns, and fragmentation profiles [11, 12]. Most of these analyses are still rather expensive and labor intensive, therefore limiting cfDNA clinical exploitation. Even more importantly the application of liquid biopsy tests has been delayed by their suboptimal sensitivity. Indeed, false negative rates remain a major issue in assays designed to detect cancer in population screens or in post-operative patients [13], and even a small fraction of cancer patients with advanced stage disease remain undiagnosed by liquid biopsy approaches [14–17]. The reasons accounting for the low or undetectable shedding behavior of certain tumors have not been elucidated. Understanding the mechanisms underlying cfDNA release could provide novel knowledge that may be applied to improve the sensitivity of liquid biopsy tests.

In comparison to the body of literature describing the clinical applications of liquid biopsy, only a few mechanistic studies have investigated the molecular basis of cfDNA release. Factors leading to DNA damage, including chromosomal instability or external agents such as radiation, chemicals, or microorganisms, may trigger the release of cfDNA [18–20]. Although the majority of cfDNA is passively shed by apoptotic and necrotic cells, active cfDNA release by dividing cells is also known to occur [21, 22]. Recent studies have shown that the fragmentation pattern of tumor-derived cfDNA differs from that of normal tissues cfDNA, with changes in fragment sizes at different regions [23–25]. Fragment sizes do not always display the ladder profile associated with apoptosis [26] or the high molecular size of necrosis [27], suggesting that secretion can contribute to shedding [28]. This is supported by the observation that alterations conferring acquired drug resistance may appear in the blood of cancer patients several months before the radiological detection of clinical relapse, suggesting the involvement of drug-refractory live cells in the DNA shedding [4, 29, 30]. It is inherently difficult to dissect the active and passive mechanisms of cfDNA release directly in the blood of cancer patients. Plasma cfDNA contains a high and variable amount of DNA derived from non-neoplastic cells especially promoted by inflammation and comorbidities, and acting as confounding factors [23, 31, 32]. We hypothesize that preclinical cancer model systems in which only neoplastic cells are present in a known number can simplify functional studies to unveil novel mechanisms of DNA shedding. For instance, a recent CRISPR screening identified genes involved in the TNF-related apoptosis-inducing ligand (TRAIL) apoptotic pathway and other genes encoding RNA-binding proteins as regulators of cfDNA release in a normal breast epithelial cell line [33]. In this work, we took advantage of a large collection of colorectal cancer (CRC) cell lines to characterize the impact of cancer cell biology parameters and molecular features on cfDNA release.

## Methods

### Aim, design and setting of the study

Colorectal tumors release variable levels of detectable cfDNA [7]. We aimed to investigate whether any cancer cell intrinsic features could be associated with release of cfDNA. To achieve this goal, we selected from a large collection of CRC cell lines, a subset of pre-clinical models recapitulating the main molecular features found in clinical samples [34, 35]. These models were assessed for cfDNA release, cell proliferation and death, and cell cycle phases. In parallel, genetic and epigenetic data were available or generated thus allowing to evaluate associations with cfDNA release.

### Cell lines

A collection of 240 CRC cell lines was employed in this study, of which 76 models were analyzed in the screening phase and the remaining 164 were exploited as a validation dataset. The majority of cell lines have previously been described [36–39] and their source and main characteristics are summarized in Additional file 2: Table S1. Cells were routinely supplemented with FBS 10%, 2 mM L-glutamine, antibiotics (100 U/mL penicillin and 100 mg/mL streptomycin), and grown in a 37 °C and 5% CO_2_ air incubator. The genetic identity of cell lines was confirmed less than 2 months from profiling experiments using the PowerPlex® 16 HS System (Promega), through Short Tandem Repeats (STR) tests at 16 different loci (D5S818, D13S317, D7S820, D16S539, D21S11, vWA, TH01, TPOX, CSF1PO, D18S51, D3S1358, D8S1179, FGA, Penta D, Penta E, and Amelogenin). Amplicons from multiplex PCRs were separated by capillary electrophoresis (3730 DNA Analyzer, Applied Biosystems). The results were analyzed using GeneMapper V5.0 software. The STR profile of each CRC cell line is reported in Additional file 2: Table S1. HCT116 CRC cells with a genetic disruption of DNMT3B and DNMT1 (DKO) were obtained from Horizon Discovery Plc. An additional small subset of commercially available pancreatic cancer cell lines, namely BXPC-3 (Cat. No ATCC-CRL-1687), CAPAN-1 (Cat. No ATCC-HTB-79), HPAF-II (Cat. No ATCC-CRL-1997), and ASPC-1 (Cat. No ATCC-CRL-1682), was obtained from ATCC for additional functional experiments.

### Basal cfDNA release protocol

For all CRC and pancreatic cancer cell lines selected in the screening protocol, a minimum of two release experiments were performed. At day 0, cells were detached from growing plates and counted via Countness II (Life Technologies). Two million live cells were plated in two 10-cm dishes, while 5∙10^5^ cells were analyzed for cell death by annexin V/propidium iodide staining (Thermo Fisher Scientific, 88–8007-74) and flow cytometry analysis (eBioscience). In parallel, a minimum of two million live cells were pelleted and fixed by ethanol 70% for cell cycle analyses. Cell cycle phases (G1, S, G2, M) were determined by flow cytometric analysis of Phospho-Histone 3 (PH3) (Abcam, ab14955). After 4 days of culture, supernatants from both dishes were collected and centrifuged at 3000* g* for 10 min, without break. Then, 10 µl of supernatant was measured using the Qubit 4 (ThermoFisher) dsDNA HS, with standards corrected with 10 µl of the matching cell culture media. cfDNA concentration was then calculated using the linear regression obtained from the media corrected standards according to manufacturer protocol. In parallel, cells were collected and analyzed for cell death and cell cycle analyses as described below.

### Flow cytometry analyses of cell biology parameters and immunofluorescence for mitotic phases

All samples were run on a BD accuri C6. A minimum of 3 × 10^4^ events were acquired for every experiment. The resulting Fcs files were exported from the flow cytometer and analyzed using FlowJo 10 (LLC). For cell death, morphological parameters were gated to exclude any cell debris. Duplets were removed using the pulse width feature and the subset of events were stratified on FL4 (APC) and FL3 (PI), to identify the different cell populations. The quarters were adjusted for every experiment in order to separate most accurately the clusters of fluorescence. For cell cycle analyses, after removal of the cell debris, duplets were removed by gating samples based on FL3 area vs. FL3 intensity. Cell cycle phases were calculated using the default FlowJo algorithm.

To measures the percentage of mitotic cells in the different phases, i.e., from prophase to telophase, cells were seeded on poly-L-lysine-coated coverslips, fixed with 2% formaldehyde, permeabilized for 10 min with 0.25% Triton X-100, and blocked for 60 min with 5% BSA in PBS. Cells were stained with anti-ß-tubulin-Cy3 Ab (Sigma-Aldrich). DNA was marked with HOECHST 33342 (Sigma-Aldrich). Cells were examined with a NIKON Eclipse Ti2 microscope equipped with epifluorescence and photographs were taken (× 60 objective) using a cooled camera device (DS-Qi2). Different mitotic phases were counted on 1000 cells per sample, in triplicate.

### Cell doubling time assay

In parallel to the cfDNA release experiments, independent assays were carried out to estimate the doubling time of each cell line. A total of 2.5 × 10^6^ living cells were labeled using the CellTrace™ Violet Cell Proliferation Kit (Thermo Fisher Scientific, C34554), according to the manufacturer’s instructions. The cell doubling time was measured for seven consecutive days by flow cytometric detection of cell trace violet incorporation.

### Annotation of cell line molecular features

Annotation of sequence variants was performed based on Whole Exome data as previously described [40]. Sample mutational status for *APC*, *TP53*, *KRAS*, *NRAS*, and *BRAF* (Additional file 2: Table S1) was determined based on presence of somatic mutations that are associated with the alteration of protein sequences (missense SNVs, nonsense SNVs, and indels). Absolute total copy number of each gene in the genome was determined as the ratio of median gene depth and the overall median depth in the whole exome. The aneuploidy score (AS), defined as the sum of the number of altered chromosome arms [41], was calculated by assigning to each arm + 1 if different from 2 copies and 0 if diploid. It ranges from 0 (all arms are diploids) to 39 (all arms are not diploids). For the score assessment, long and short arms for each non-acrocentric chromosome and only long arms for chromosomes 22, 21, 15, 14, and 13 were considered. The microsatellite instability (MSI) status was evaluated by using the MSI Analysis System Kit (Promega) according to the manufacturer’s protocol. The analysis requires a multiplex amplification of seven markers including five mononucleotide repeat markers (BAT-25, BAT-26, NR-21, NR-24, and MONO-27) and two pentanucleotide repeat markers (Penta C and Penta D). The microsatellite status of each CRC cell line is reported in Additional file 2: Table S1 as MSS (microsatellite stability) or MSI (microsatellite instability). The products were analyzed by capillary electrophoresis in a single injection using ABI 3730 DNA Analyzer capillary electrophoresis system (Applied Biosystems). The results were analyzed using GeneMapper V5.0 software (Life Technologies). Annotations of CMS transcriptional subtypes (CMS1, CMS2, CMS3, or CMS4) and CRIS transcriptional subtypes (CRIS-A, CRIS-B, CRIS-C, CRIS-D, or CRIS-E) were based on RNA seq data as defined by Eide et al. and Isella et al., respectively [42, 43].

### DNA methylation microarray experiments

DNA methylation data were previously obtained using the Infinium HumanMethylation450 BeadChip array [14] or recently analyzed with the Infinium MethylationEPIC BeadChip microarray. DNA samples (500 ng) were treated with sodium bisulfite using the Zymo EZ-96 DNA Methylation-Lightning Kit (Zymo Research, CA, USA). The bisulfite-converted DNA was then processed and hybridized onto the BeadChips according to manufacturer instructions (Illumina Infinium HD Assay Methylation Protocol Guide).

After single base extension and fluorescent staining, the BeadChips were imaged with the Illumina iScan high sensitivity scanner (Illumina Inc., San Diego, CA), and raw data recorded as *.idat files.

### Bioinformatic analyses of methylation microarray data

HM450 and EPIC raw data (IDAT files) were processed with a similar workflow using the minfi R Bioconductor package and corresponding annotation packages [44]. In each sample, probes with detection *p*-value > 0.05 were masked in the RGChannelSet. In addition, cross-reactivity probes and probes matching SNPs at the target CpG site were filtered using previously published lists [45, 46]. Then, methylation and unmethylation signals were obtained for each CpG site using the preprocessnoob function and β-values were computed [47]. Results obtained in all samples were subsequently merged in a single β-matrix, thus keeping only CpG sites targeted by both HM450 and EPIC platforms (452,832 CpG sites). Furthermore, CpG sites that are located on sex chromosomes or chrM were excluded (13,836 CpG sites). We performed PCA to rule out the potential batch effects due to slide or position (data not shown). The annotation of probes differentially methylated between high and low releasers was retrieved from the Infinium HumanMethylation450k manifest file. Genes were then associated with hallmark gene sets from MsigDB collection by using the msigdbr R package [48].

For the annotation of DNA methylation-based subtypes, we referred to a previously published list of 318 probes that showed significantly higher DNA methylation level in both CIMP-H and CIMP-L tumors with respect to non-CIMP tumors (CIMP-associated probes) [49]. Starting from the β-matrix obtained as described above, 284 out of 318 CIMP associated probes were selected by excluding those containing missing values. Then, we performed unsupervised clustering using recursively partitioned mixture model (RPMM) with max level = 2 and finally we associated the resulting clusters to CIMP classes based on their median DNA methylation level (CIMP-H, CIMP-L, CIMP3, and CIMP4, for high to low median DNA methylation level).

### cfDNA release protocol from organoids

The characteristics of 12 CRC patient-derived organoids (IRCC105_A_PDO, IRCC105_C_PDO, IRCC105_E_PDO, IRCC107_A_PDO, IRCC10_A_HL, IRCC120_PDO, IRCC125_A_PDO,IRCC131_PDO,IRCC148_A_PDO,IRCC150_A_PDO,IRCC161_A_PDO, IRCC166_PDO, IRCC174_A_PDO) are summarized in Additional file 3: Table S2. PDOs were suspended in a 3D extracellular matrix substitute (Matrigel®, Corning) and dispensed in the 12-well plates as 200-µL droplets. After matrix polymerization (20 min at 37 °C), the droplets were overlaid with pre-warmed Dulbecco’s modified Eagle medium/F12 supplemented with penicillin–streptomycin, 2 mM L-glutamine, 1 mM n-Acetyl Cysteine, 1X B27, 1X N2, and 20 ng/ml EGF (Sigma-Aldrich).

For cfDNA analyses, at baseline (day 0) PDOs were plated in Matrigel coated 12-well plates with a medium supplemented with 2% Matrigel. After 4 days, supernatants were collected and centrifuged at 3000* g* for 10 min, without break. Then, 10 µl of supernatant was measured using the Qubit 4 (ThermoFisher) dsDNA HS. The cfDNA concentration was calculated by normalizing the Qubit value in relation to the number of cells.

### Patient-derived xenografts (PDXs)

The mutational, transcriptional, and methylation profiles of PDXs presented in this paper represent a subset of a larger xenograft cohort, which is described in [50], collected and characterized in the Translational Cancer Medicine laboratory in Candiolo Cancer Institute FPO-IRCCS. Research involving human specimens was performed in accordance with the Declaration of Helsinki. The characteristics of these 490 PDXs are summarized in Additional file 4: Table S3. A dataset of 490 PDX methylation profiles was obtained using Illumina MethylationEPIC (v1) bead chip. Raw data was processed with the Minfi package (v1.32.0), using the function *preprocessNoob* [47]. To account for possible methylation signals originating from the murine infiltrate, we removed probes known to specifically map on the mouse genome. To this end, we combined two lists of murine-specific probes, obtained from Gujar et al. and Needhamsen et al. which resulted in removal of 22,537 probes [51, 52]. Six samples were removed in quality control (median intensity < 10.5); 64,361 probes were removed for detection *P* > 0.01. Probes mapping on X and Y chromosomes (19,627), on SNPs (8423), and on multiple loci on the genome (43,177) were also removed as gold standard filtering [53]. CIMP annotation for this dataset has been obtained using the CIMP-general (CIMP-H & CIMP-L) markers panel from Hinoue et al. [49].

### Pharmacological treatment

Five CRC cell lines were treated with decitabine (DAC, 5′-Aza-2’-deoxycytidine) that was purchased from Sigma-Aldrich, diluted in DMSO and added at 1 nM concentration. After 4 days of treatment, without significant changes in cell viability, the media was refreshed and cell lines were seeded for cfDNA screening for additional 4 days in the absence of drug. The same treatment protocol was applied to BXPC-3, CAPAN-1, HPAF-II, and ASPC-1 pancreatic cancer cell lines using experimentally determined sub-lethal concentrations of DAC.

### Assay for transposase-accessible chromatin sequencing (ATAC-seq) analysis

HCT116 and HCT116 DKO cells were detached from growing plates, at baseline (day 0). One million living cells were plated in 10-cm dishes and cultured for 3 days. For ATAC-seq analysis, cells were cryopreserved in 1 ml of serum-free cryopreservation media (Bambanker, Nippon Genetics Europe) containing 1 × 10^6 cells in biological triplicate. The ATAC-seq analyses were performed following manufacturer instructions (Diagenode ATAC-seq kit, Cat. No. C01080002). Raw FASTQ files were initially trimmed using the Trim Galore tool and mitochondrial reads were discarded using Xenome [54]. The samples were aligned to hg38 genome using BWA-mem, duplicates were marked using Picard Tools MarkDuplicates and only deduplicated properly paired reads were selected for further analysis. In order to account for Tn5 shift, all positive strand reads were shifted by + 4 bps and all negative strand reads were shifted by − 5 bps. Peak calling was then performed individually for each sample using MACS2 [55] and peak scores for each sample were normalized to a “score per million” for read depth variations [56]. Next, the peak summits were extended by 250 bp on either side to a final width of 501 bp, filtered by the ENCODE hg38 blacklist [57], and filtered to remove peaks that extend beyond the ends of the chromosomes. Overlapping peaks for each condition (HCT116 WT and DKO) were handled to generate consensus peak sets [56]. The number of fragments overlapping the consensus peak set were computed using SAMtools depth resulting in an insertion counts matrix [56]. Next, DESeq2 was used to perform differential chromatin accessibility analysis. Adjusted *p*-values were calculated using the Benjamini–Hochberg (BH) method and only regions with adjusted *p*-value of less than 0.05 were considered [56].

### Transcriptomics analysis

RNA from cells was extracted using the Maxwell® RSC miRNA Tissue Kit (Promega, Cat# AS1460) and RNA-seq analysis was performed using TruSeq® Stranded mRNA Library Prep (Illumina, cat# 20020594), according to the manufacturer’s protocols. RNA-seq reads were aligned to hg38 using STAR aligner and subsequently RSEM was used for transcript and gene quantification and GENCODE v44 as gene annotation. Then, starting from RSEM genes results, robust FPKM values were computed using DESeq2. Two conditions (HCT116 WT and DKO) were compared. Independent filtering was applied using the results function, and adjusted *p*-values were calculated using the BH method. Coherently with other analysis, adjusted *p*-values of less than 0.05 were considered.

### Whole genome sequencing of nuclear and supernatant DNA

NGS libraries for DNA released in the supernatant of CRC cell lines were prepared starting with 2 µg of DNA and processed with Illumina TruSeq DNA PCR-Free kit (Illumina Inc., San Diego, CA, USA). In order to maintain the precise fragment distribution, we introduced some modifications to the manufacturer’s protocol. In particular, any fragmentation step has been avoided, as well as bead-based clean up steps have been modified with the aim to preserve all short fragments. Quality of final libraries has been assessed using the 2100 Bioanalyzer with a High-Sensitivity DNA assay kit (Agilent Technologies, Santa Clara, CA). Equal amounts of DNA libraries were pooled and sequenced on NovaSeq6000 (Illumina Inc., San Diego, CA, USA). Fastq files were analyzed as previously reported [58, 59], and the identity of all cell lines was determined using unique allelic profiles based on Single Nucleotide Polymorphism Identification (SNP_ID) annotated using the dbSNP version 153. Only alleles with a fractional abundance above 30%. SNP_ID were considered matched when the fractional abundance between two profiles exceeded 95% [60]. Mutational calling was performed as previously reported [58, 59].

## Statistics

Statistical analyses were performed using GraphPad Prism software. All data are presented as either mean ± S.D. or ± S.E.M. (as indicated in figure legends). To determine statistical significance for cfDNA values in relation to analytical variables, we applied nonparametric tests because the data did not follow a Gaussian-like distribution. Single-variable Spearman correlation was performed between cfDNA and cell doubling time, cell cycle, cell loss, or aneuploidy score. In all other cases, the Mann–Whitney test was performed. Symbols for statistical comparison are *p < 0.05; **p < 0.01; ***p < 0.005; ****p < 0.001.

## Results

### cfDNA release shows large inter-cell variability

**Fig. 1:**
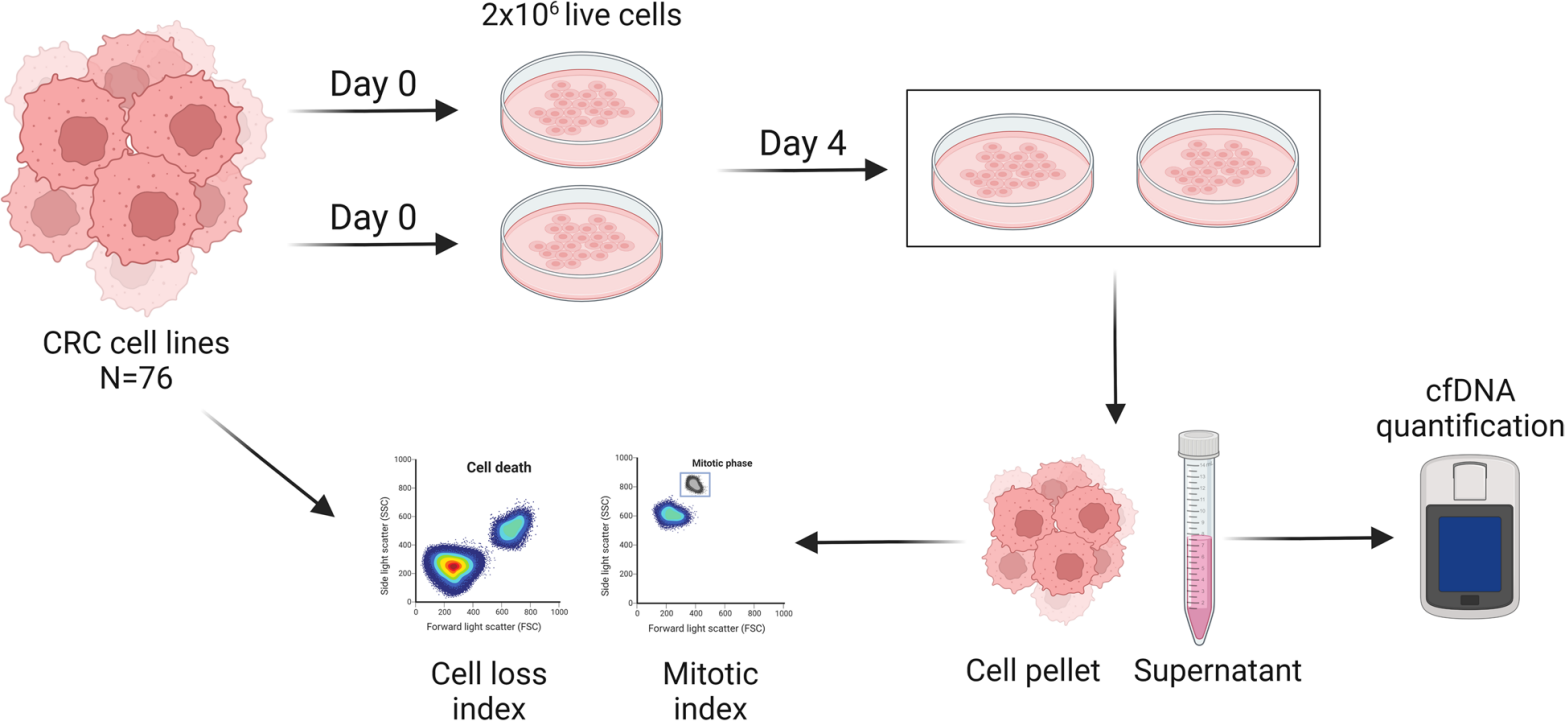
Workflow of cfDNA release detection protocol. The release of cfDNA was measured in a collection of 76 CRC cell lines including patient-derived primary models. Two million live cells were seeded in duplicate, and the supernatant was collected for cfDNA quantification after 4 days in culture without changing the medium. In parallel, the cell pellet was analyzed for cell cycle and cell death parameters

**Fig. 2:**
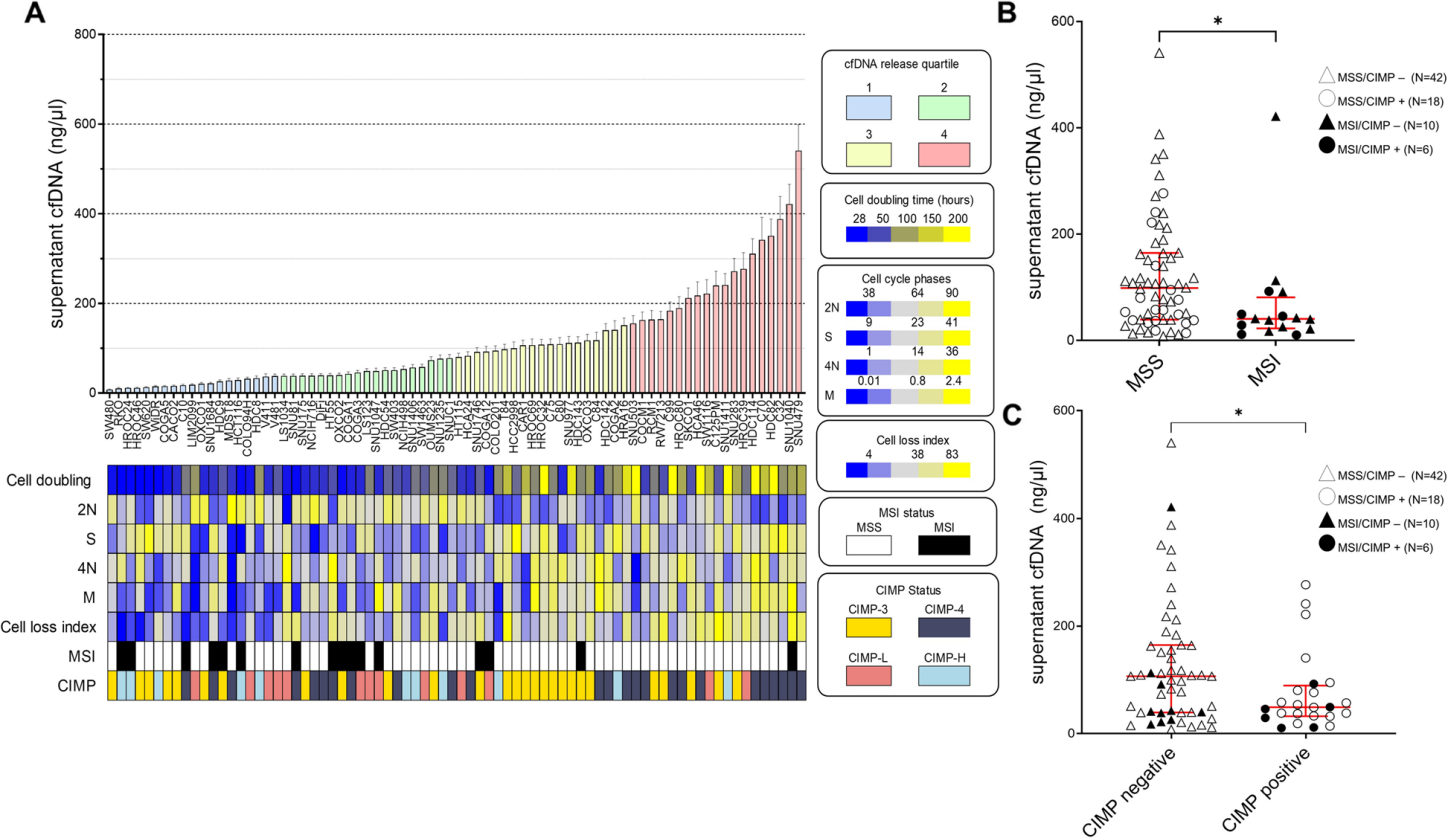
Association of cfDNA release with biological parameters and molecular features in CRC cell lines. A Histograms indicate the amount of cfDNA (ng/µl) detected in the supernatant of 76 CRC cell lines after 4 days in culture. cfDNA values detected by Qubit were normalized on the number of cells at the final day of each experiment. Data points and error bars indicate mean + SD. The column color code groups the cell lines according to the quartiles of cfDNA release. The cell doubling time was measured by fluorescent analysis of cell trace violet incorporation. Cell cycle phases (2N, S, 4N, M) were determined by flow cytometric analysis of Phospho-Histone 3 (PH3). The cell loss index (apoptotic and necrotic cells) was calculated by annexin V/ propidium iodide staining. Annotated molecular features depicted in color boxes indicate Microsatellite Instable or Stable status (MSI or MSS); aneuploidy score based on NGS data as defined by Taylor et al. [36]; CIMP classification based on four methylation classes (CIMP-1, CIMP-2, CIMP-L, CIMP-H) according to Hinoue et al. [42]. B Comparative analyses of cfDNA values in CRC cell line groups based on MSI and MSS status was performed using the non-parametric Mann Whitney test (* *p* < 0.05). C Comparative analyses of cfDNA values in CRC cell lines with grouped methylation classes labeled as CIMP negative (CIMP classes 1 and 2) or CIMP positive (low and high CIMP classes) was performed using the non-parametric Mann Whitney test (*p < 0.05)

To identify cancer cell intrinsic features associated with cfDNA release, we exploited a large collection of 240 CRC cell lines, most of which were previously reported (Additional file 1: Fig S1) [36–39]. We selected a panel of 76 CRC models representative of the main molecular subtypes occurring in clinical samples. As depicted in Fig. 1, evaluation of cfDNA release was performed at a standardized arbitrary time point after seeding recently thawed cells in their recommended culture media. The raw cfDNA values in the supernatant were normalized by the number of cells at the end of the assay to account for samples with different proliferation kinetics. Surprisingly, our screening revealed a wide dynamic range of cfDNA shed by CRC cells in culture with values between 7.8 and 540.8 ng/µl (Fig. 2A, Additional file 5: Table S4). We investigated if the genomic features in the supernatant cfDNA were in line with the nuclear DNA by performing WGS of both intracellular DNA and supernatant cfDNA in a representative subset of eight CRC cell lines. By analyzing genomic positions annotated as common single-nucleotide polymorphisms (dbSNPs) version153, we found more than 95% similarity between the supernatant cfDNA samples and their matched nuclear DNA samples (Additional file 6: Table S5). We also found that 99.67–99.99% of the reads from intracellular DNA and supernatant cfDNA aligned with chromosomal DNA, respectively. At least in the small set of cell lines analyzed for this purpose (HCT116, HCT15, C75), mitochondrial reads accounted for only 0.220 ± 0.151% (mean ± SD of the three cell lines) of intracellular DNA and 0.012 ± 0.012% of supernatant DNA. Therefore, in our setting, the contribution of mitochondrial DNA to the overall amount of cfDNA appears negligible (Additional file 7: Table S6).

### The release of cfDNA is influenced by cell proliferation and cell death-related parameters

We found that the slowest cycling cells released more DNA, as shown by the correlation between cell doubling time and cfDNA amount (*r* = 0.72; ****p < 0.001, Fig. 2A and Additional file 1: Fig S2). To corroborate this evidence, we analyzed cell cycle phases, and we found that the levels of cfDNA were significantly anti-correlated with the percentage of cells in phase 2N (rho =  − 0.31, **p < 0.005). On the other hand, cfDNA quantity showed a positive correlation with the percentage of cells in phase S (rho = 0.24, *p < 0.05), in phase 4N (rho = 0.27, *p < 0.05) and particularly in the mitotic (M) phase (rho = 0.42, ***p < 0.001). Further evaluation of the M phase in representative cells from the different cfDNA release quartiles showed enrichment in the prophase/metaphase stages in the highest quartile (Additional file 1: Fig S3). We then reasoned that, in addition to dividing cells, also dead or dying cells could play a major role in cfDNA release. A cell loss index was calculated by experimentally assessing the number of apoptotic and/or necrotic cells that contributed to cfDNA release in the supernatant. As expected, the cell loss index was significantly correlated to cfDNA quantity (rho = 0.55, **** p < 0.001) (Fig. 2A, Additional file 1: Fig S2).

All together, these observations suggest that higher levels of cfDNA release are associated with both slower cell cycling and increased cell death.

### The release of cfDNA is associated with CRC molecular features

Next, cfDNA release values were associated with molecular features such as MSI phenotype, aneuploidy score, and methylation classes (CIMP). We found that the MSI phenotype was negatively associated with cfDNA release in CRC cell lines (*p < 0.05; Fig. 2A, Additional file 1: Fig S2), recapitulating what has recently been reported in patients [61].

A trend towards an increased aneuploidy score was found in cells shedding more cfDNA (rho = 0.22, *p* = 0.05; Additional file 1: Fig S2). We then investigated the impact of the epigenotype by classifying cell lines according to Hinoue and colleagues [49]. Lower cfDNA release was observed in CIMP positive (Low and High) cells versus CIMP negative (CIMP3 and CIMP4) samples (*p < 0.05; Fig. 2B). These results suggest that the phenotype defined by high level of methylation and normal ploidy such as MSI would release less cfDNA; on the other hand, the phenotype with chromosomal instability caused by hypomethylation may lead to higher cfDNA release.

### Derivation and validation of a methylation signature to predict cfDNA release in CRC cell lines

**Fig. 3:**
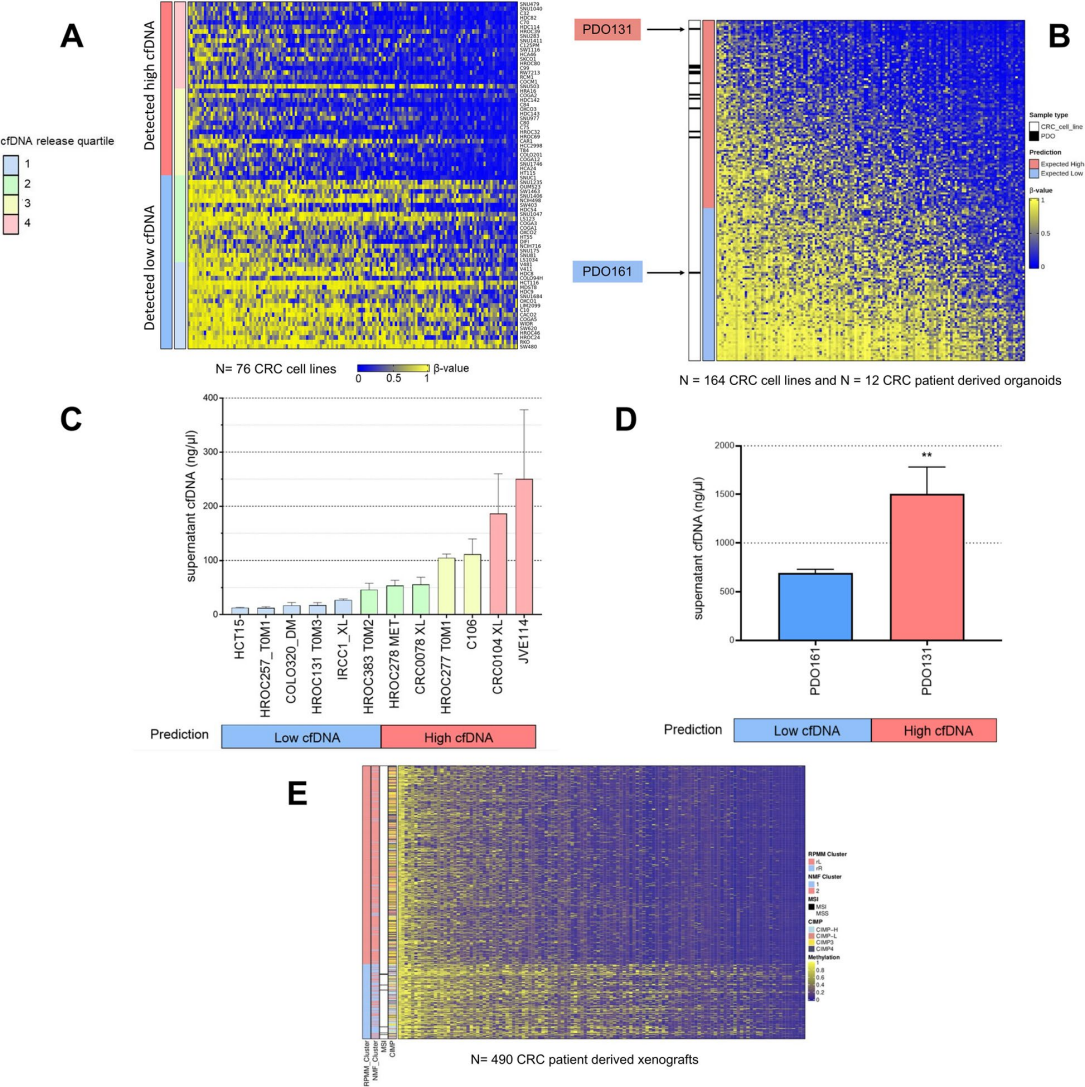
Derivation and validation of a methylation signature to predict cfDNA release in CRC cell lines, patient-derived organoids and patient-derived xenografts. **A** Heatmap depicting β-values of individual CpG Islands differentially methylated between cell lines experimentally classified in two groups (high *vs* low cfDNA) based on the amount of cfDNA shed in the supernatant. High cfDNA and low cfDNA screening classes include cell lines distributed in the top two or bottom two quartiles, respectively. Rows in the heatmap indicate samples sorted by cfDNA release values, while columns represent probes sorted by mean methylation level across samples.** B **Differentially methylated probes between high and low cfDNA releasing cells were applied to a validation dataset of 164 CRC cell lines and 12 PDOs to predict cfDNA release classes. Heatmap rows rank all samples from expected high to expected low cfDNA based on silhouette width values. Columns indicate probes sorted by mean methylation level across all samples. **C** Evaluation of cfDNA release in the supernatant of the indicated CRC cell lines, including patient-derived models (HROC257_T0M1, HROC131_T0M3, IRCC1_XL, HROC383_T0M2, HROC278_MET, CRC0078_XL, HROC277_T0M1, CRC0104_XL), chosen among those predicted to be high or low releasers based on the heatmap shown in panel **B**. The color code of each bar is based on cfDNA screening quartiles highlighted in Figure 2A. Columns and error bars indicate mean + SD.** D** Evaluation of cfDNA release in the supernatant of the indicated CRC patient-derived organoids, chosen among those predicted to be high or low releasers based on the heatmap shown in right panel **A**. Columns and error bars indicate mean + SEM.** E **Heatmap depicting β-values of individual CpG Islands (125/145, due to the filtering of probes that could hybridize to the mouse genome, see Methods) of the signature in 490 CRC PDXs, divided in the two groups identified by the methylation classifier. Molecular annotations and the clusters identified by NMF with *k*=2 are shown on the left. MSI or CIMP positive samples are enriched in the low cfDNA release group (Fisher’s test, all *** *P* < 0.005)

Intrigued by the above findings, we decided to investigate more thoroughly the impact of the DNA methylation profile on cfDNA release. We performed a linear regression modeling using methylation microarray probes to stratify the previously screened 76 cell lines by quartiles of cfDNA values. Using methylomes from cell lines falling in the first (*n* = 19) and fourth (*n* = 19) quartiles of detected cfDNA values, we identified a subset of 145 differentially methylated probes that were significantly associated with cfDNA release (FDR < 0.01). Annotation of the genes associated with differentially methylated gene promoter regions in high versus low releasers did not reveal any specific pathway enrichment (Additional file 8: Table S7). Cells with higher levels of methylation at these 145 loci displayed lower cfDNA shedding and the signature discriminated high from low releaser samples (Fig. 3A, Additional file 9: Table S8). We then applied this methylation signature to predict cfDNA release in an independent collection of 164 CRC lines and 12 CRC patient-derived organoids (PDOs) for which methylomes had been previously reported [14] or purposely generated (Fig. 3B, Additional file 10: Table S9). By this analysis, a total of 79 and 97 CRC models were predicted to be low and high releasers, respectively. When our methylome signature was applied to these CRC models, samples predicted as low cfDNA releasers were strongly enriched in CIMP-high (hypergeometric test *p*-value = 5.3e − 09) and MSI status (hypergeometric test *p*-value = 5.7e − 09). From this validation collection of preclinical models, we selected 12 CRC lines predicted to be high or low releasers. To increase the translational relevance of this dataset, eight of these models were primary cell lines recently established from CRC patients. Cell lines employed as a validation dataset were subjected to the previously described protocol depicted in Fig. 1 to experimentally detect cfDNA in the supernatant. Similarly to the screening results (Fig. 2A), we observed again a large variability of cfDNA amounts among validation cell lines (Fig. 3C). Cancer cell lines predicted to be low releasers according to the methylation classifier displayed decreased supernatant cfDNA compared to models predicted to be high releasers (**p < 0.005). By this approach, all but one organoid were predicted to be high cfDNA releasers (Fig. 3B). Two of these models (one predicted to be a high releaser CRC131, and the one predicted to be low releaser CRC161) were then experimentally validated. Our results showed that the organoid predicted to be low releaser sheds significantly less cfDNA than an organoid predicted among the high releasers (Fig. 3D).

In order to have in vivo validation for cfDNA measurements and for improving the clinical relevance of our work, we analyzed the methylome profiles of a previously established collection of 490 CRC patient-derived xenografts (PDXs). When our methylome signature was applied to the PDXs, all MSI cases clustered in the group of samples predicted as low cfDNA releasers (Fig. 3E). CIMP-high tumors were enriched in samples predicted as low cfDNA releasers (*p < 0.005).

### Genetic or pharmacological depletion of DNA methylation promotes cfDNA release

**Fig. 4:**
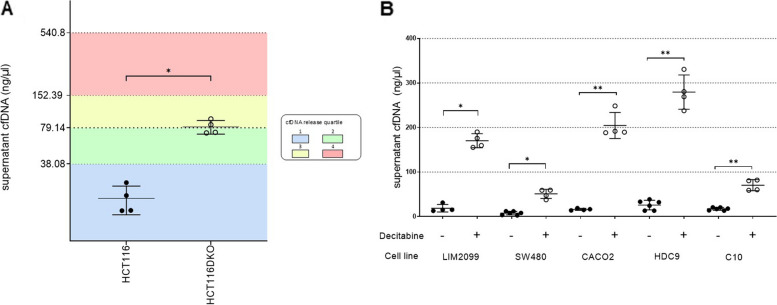
Genetic and pharmacological depletion of DNA methylation stimulates cfDNA release. **A** cfDNA release was increased in the supernatant of CRC cells with inactivation of DNA Methyltransferases 1 and 3B (HCT116DKO) compared to their parental counterpart (HCT116). Circles indicate individual replicates. Unpaired test with Welch’s correction (* *p* < 0.05). **B** cfDNA shedding was increased in the supernatant of low releasing CRC cells exposed to non-toxic concentrations (1 nM) of the demethylating agent decitabine. Statistical significance: * *p* < 0.05, ** *p* < 0.01

Our data indicated an association between DNA methylation status and cfDNA release. To functionally characterize the impact of DNA methylation on cfDNA release, we undertook two functional approaches. We first exploited the HCT116 DNMT1/DNMT3B knockout (HCT116 DKO) cell line which lacks the enzymes establishing and maintaining the methylation patterns, leading to an almost completely demethylated genome as previously described [62]. We found that HCT116 DKO released significantly more cfDNA as compared to the parental cell line (***p < 0.001; Fig. 4A). Consistently, we observed that the supernatant of HCT116 DKO contained more abundant copies of the KRAS G13D mutant allele compared to parental cells (Additional file 1: Fig S4). As a second functional approach, we treated five low releaser models with the demethylating agent decitabine at a 1 nM concentration which has been shown to deprive cells from methylation without impacting cell survival [63]. We found that decitabine significantly increased the amount of cfDNA shedding in all tested cell lines (Fig. 4B). This suggests that global changes in the methylation pattern can promote cfDNA release in CRC models. To generalize our findings to another tumor type, we applied decitabine to four pancreatic cancer cell lines and we observed an increase of cfDNA in the supernatant also in these models (Additional file 1: Fig S4).

### cfDNA release is associated with increased chromatin accessibility and fragmentation

**Fig. 5:**
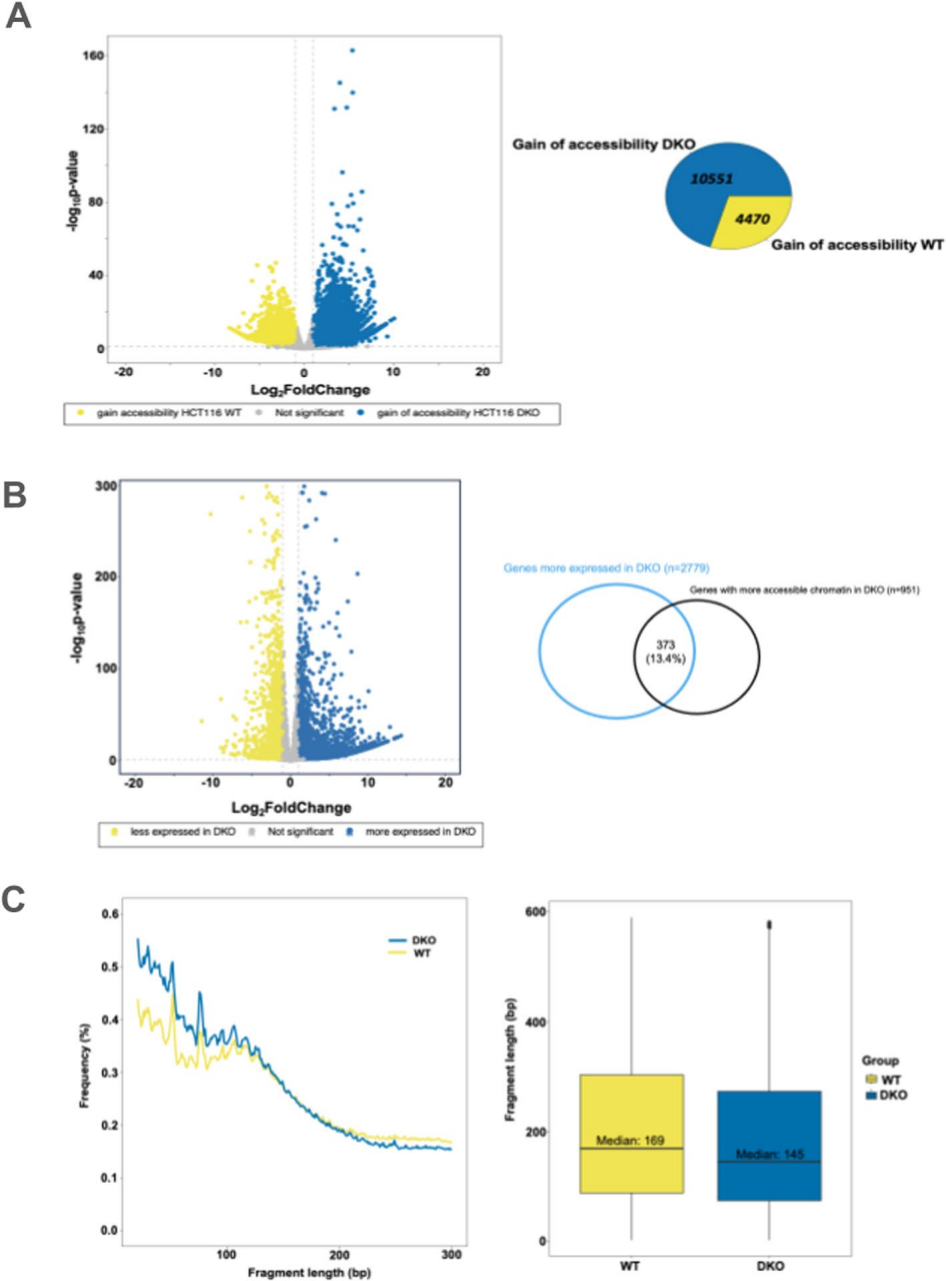
Association between cfDNA release and chromatin accessibility. **A **Differential chromatin accessibility analysis between HCT116 DKO and their parental WT counterpart. **B **Differential gene expression analysis between HCT116 DKO and their parental WT counterpart**. **Out of the 2779 genes upregulated in DKO in comparison to WT cells, 373 showed increased chromatin accessibility in their promoter. **C **Fragment length distribution of intracellular DNA from HCT116 WT and DKO cells analyzed by ATAC-seq. Statistical significance: **** *p* <0.0001

ATAC-seq was employed to characterize the chromatin accessibility of intracellular DNA of the HCT116 DKO cells in comparison to their parental cells (HCT116 WT). As expected, we observed a genome-wide rewiring of chromatin accessibility by identifying 10,551 gain of accessibility events in DKO cells, in comparison to 4470 regions that were more accessible in WT (Fig. 5A).

RNA-seq analysis revealed a total of 4455 genes differentially expressed between isogenic pairs. Among these, 1676 genes were downregulated, and 2779 genes were upregulated in DKO cells in comparison to WT cells (L2FC > 1 and adjusted *p* < 0.05, Wald test with BH correction). Of the 2779 upregulated genes, 373 (13.4%) exhibited increased chromatin accessibility in their promoters (Fig. 5B). This overlap was higher than the overlap obtained by chance when the same number of genes with random promoter accessibility (iterating 10 times) was intersected against the set of more expressed genes in DKO (5.6 ± 0.4%).

We also analyzed the fragmentation pattern of DNA associated with differentially accessible chromatin regions. We observed a statistically significant enrichment of shortened intracellular DNA fragments in HCT116 DKO in comparison to WT cells (****p < 0.0001; Wilcoxon rank-sum test) by the analysis of ATAC-seq data (Fig. 5C).

It is plausible that specific nucleases could more easily cut DNA within nucleosomes in regions with higher nucleosome accessibility.

## Discussion

Recent technological advances have allowed the isolation and analyses of circulating nucleic acids in different physiopathological conditions beyond oncology, including non-invasive prenatal screening, autoimmune diseases, organ transplantation, and chronic liver disease [64, 65]. In oncology, analysis of cfDNA in blood can be applied for early cancer detection, monitoring of tumor burden as well as to study tumor heterogeneity and mechanisms of drug resistance [66–68]. The amount of cfDNA shed by human cancers has consistently been correlated to tumor stage. However, some advanced tumors release minimal to undetectable amounts of cfDNA, defining the concept of non-shedders; while some early malignancies are clearly revealed by liquid biopsy [14, 17, 31]. Some of the discrepancies in cfDNA release among tumors with different histology can be attributed to anatomical and vascular barriers. However, highly variable levels of cfDNA have also been found in patients with tumors of the same stage and histopathological origin, raising questions about the factors affecting cfDNA release [10]. We believe that basic functional studies should complement clinical observations and help elucidate the mechanisms of cfDNA shedding. We tested the hypothesis that cancer cell intrinsic features could influence the amount of cfDNA release. We exploited a large collection of cancer cell lines sharing colorectal origin, to experimentally quantify the cfDNA release in the supernatant. The preclinical models employed for the large initial screen were selected to mirror the main molecular and pathological subtypes observed in patients. Surprisingly, cfDNA values shed by CRC cell lines spanned over nearly three logarithms of concentration, thereby reproducing the variability in cancer patients [69]. Experiments carried out under controlled laboratory conditions allowed measuring cell biology parameters in preclinical models, such as cell doubling time, cell cycle phases and cell death that are not easily determined in patient samples. Previous works also exploited cancer cells in culture to study the basic biology underlying the process of cfDNA shedding in the supernatant [70]. However, these studies investigated in detail only a limited number of cell lines of different histology. Our work represents the first large atlas depicting cfDNA of CRC origin in relation to the intrinsic variability of different cell biology parameters. We found that slower cycling cells release more cfDNA in the supernatant which is associated with an increased fraction of cells in the mitotic phase. Conversely, high turn-over of the cells, defined by fast growth, was previously suggested to be the origin of increased cfDNA values (reviewed in [71]). Interestingly, a correlation has been found between the staining of the proliferation marker Ki67 in patient samples and cfDNA release in breast and lung cancer but not in CRC [72, 73]. In accordance with previous studies, we found a clear association between increased cell death and cfDNA release reporting that apoptotic and necrotic cells contribute to cfDNA in the circulation [74].

We noted that high cfDNA releaser cells often displayed a concomitantly high cell loss index as well as an increased percentage of cells in the M-phase with enrichment in the prophase to metaphase in representative lines, suggesting the presence of chromosome segregation [75]. The co-occurrence of these factors suggests a possible link between cfDNA release, aneuploidy, or mitotic catastrophe often associated with large chromosomal DNA damage [21].

Among the molecular features associated with cfDNA, we found that low releaser cancer cell lines were enriched for MSI and CIMP positivity, while aneuploid and CIMP negative cells tended to release higher levels of cfDNA. Despite the fact that levels of plasma cfDNA are often sufficient to detect MSI status in metastatic CRC patients [76], a recent study has shown that MSI-high status was significantly associated with a decreased probability of detecting tumor derived circulating DNA in a large cohort of surgically resected CRC patients [69].

The MSI status is often associated with *BRAF* mutations and a positive CIMP phenotype in CRC samples [77–80]. The abovementioned study on resected CRC patients found an impact on cfDNA of concomitant *BRAF* mutations within the MSI positive subgroup, but it did not provide methylome classification [81]. The small number of cell lines displaying overlap between MSI status and *BRAF* mutations prevented us from performing subgroup analysis of experimental data. Nevertheless, we were able to perform correlation analyses between cfDNA in the supernatant and methylome classification. Despite the observation that MSI and CIMP positivity were enriched in low cfDNA releasers, these molecular variables were not sufficient to fully explain the variability in the release of cfDNA in our collection of CRC models. The unexpected association between methylation classes and cfDNA prompted us to exploit methylome data to derive a methylation signature that could efficiently predict cfDNA release. By applying this classifier to methylome data of an independent dataset of CRC preclinical models, we were able to successfully predict the releasing behavior by individual primary cell lines and patient-derived organoids. Methylome signatures have been described to detect the presence or estimate the quantity of tumor-derived DNA in plasma cfDNA of cancer patients [82]. However, we are not aware of studies that have derived a methylation-based signature to predict the intrinsic propensity of cfDNA release. Although we were not able to provide direct patient measurements, we applied our release signature to methylomes of a very large PDX dataset. We found that tumors classified as CIMPs or MSI high were predicted as low releasers of cfDNA. In the future, our methylation signature should be tested prospectively in CRC patients enrolled in liquid biopsy trials. If validated, it might have clinical implications in the prediction of low cfDNA shedding tumors and the identification of cfDNA false negative assays in individuals with cancer.

Aberrant methylation patterns occur very early and are considered a hallmark in the carcinogenesis process with global hypomethylation followed by altered hypermethylation of specific DNA regions [83, 84]. We hypothesized that the functional modulation of methylation patterns could impact the propensity of cancer cells to release cfDNA. We found that methylation depletion was associated with increased cfDNA in the supernatant of a genetically engineered cancer model. We sought to corroborate this finding by pharmacological demethylation treatment. All tested CRC cell lines released significantly more cfDNA when treated with a short course of decitabine at a concentration known to affect DNA methylation without inducing cytotoxicity. We replicated these observations in a panel of pancreatic cancer cell lines indicating that the impact of demethylation on cfDNA is not dependent on histology. Demethylating agents are approved for hematological malignancies and their use in patients with solid tumors is limited to clinical trials [85]. In this regard, the ORIENTATE clinical trial (NCT05360264) is designed to test the efficacy of decitabine in selected pancreatic adenocarcinoma patients. Plasma samples from patients enrolled in this trial may be collected to test whether demethylating agents can improve the release of cfDNA from solid tumors. The potential limitation to this model is that the decitabine treatment may induce an increase in release of cfDNA by normal cells.

Our finding that enhancing demethylation promotes cfDNA release leaves open questions on the underlying molecular mechanisms, which we attempted to partially explain by performing ATAC-seq and WGS in the cells knocked out for DNA methyltransferases in comparison with the wild type. We found that the cfDNA release is associated with increased nucleosome accessibility and higher cfDNA fragmentation. Our observation is indeed consistent with prior work reporting that the length of cfDNA fragments is shortened in the presence of lower DNA methylation levels, which presumably impacts higher nucleosome accessibility that in turn favors cutting within the nucleosomes by specific nucleases [23].

Our work presents some limitations. As we solely relied on microarray technology for methylation analysis, in the future we wish to exploit advanced next-generation sequencing methods for epigenetic analysis. We did a partial investigation of intrinsic parameters of cfDNA such as fragmentation and nucleosome accessibility. Although these analyses provided initial mechanistic clues on cfDNA variability, we believe that in-depth analysis is beyond the scope of this work. Differently from other cfDNA studies on cell lines, we did not assess the release kinetics. Due to the complexity of testing a large number of models, we carefully selected a single time-point for supernatant collection to ensure optimal culture conditions and avoid over-growth. We limited the analysis to cell biology parameters including proliferation, cell cycle and cell death, but we did not investigate the implications of cellular metabolic pathways. Interestingly, a previous study proposed that the pattern of cfDNA could be dependent on the glycolytic activity of cancer cell lines [86]. Future metabolomics profiling of our large CRC collection might allow correlative analyses with cfDNA.

Another caveat of our cell culture approach is that we did not assess the impact of the local tumor microenvironment. The crosstalk of cancer cells with fibroblasts, endothelial, and immune cells could influence the release of cfDNA [4, 70], and future co-culture experiments could shed light on this aspect. We acknowledge that our initial screening was performed in conventional 2D cell culture conditions and did not take into account the presence of factors or forces of the extracellular matrix [87]. For instance, it would be interesting to investigate how stiffness can influence cfDNA release by growing cells in different mixed collagen-matrigel matrices. We also note that we measured cfDNA in the supernatant of cells cultured at conventional laboratory gas and media conditions, which may not recapitulate the oxygen and nutrient concentrations found in the tumor microenvironment. In this regard, perhaps the major limitation of our in vitro work is the lack of vascular barriers, blood supply, and clearance that are typical of the human body. To address these issues, others had proposed to employ xenograft models of human tumors to study cfDNA [88]. However, this in vivo approach also has caveats due to the lack of vascularization in subcutaneous xenografts, and the low volume of blood circulating in mice.

## Conclusions

This is the first work dissecting the release of cfDNA in a large dataset of CRC preclinical models. This study brings some clinically relevant insights potentially describing tumor intrinsic features that contribute to explain the variability of cfDNA amounts seen in CRC patients.

## Supplementary Information

Additional file 1: Fig S1.Annotation of 240 CRC cell lines**. **Fig S2. Association between cfDNA values and biological parameters or molecular features. Fig S3. Mitotic phases in representative CRC cells from the different cfDNA release quartiles. Fig S4. Quantification of KRAS mutant allele copies for HCT116 (parental and DKO) in supernatant cfDNA extracted from different volumes of cell supernatants. Fig S5. Pharmacological depletion of DNA methylation stimulates cfDNA release in pancreatic cancer cell lines.

Additional file 2: Table S1. Characteristics of CRC cell lines. (a-b) Annotation of the 240 CRC cell lines. For each cell line we report: (1) Cell line name; (2) Primary tumor site; (3) Source: established in our lab (from surgical samples or PDXs), obtained through a collaboration or purchased from an international cell line bank; (4) Supplier ID: detail about the cell line origin (the scientist with whom the collaboration was established or name of the international cell line bank); (5) Microsatellite status: microsatellite stable (MSS) or instable (MSI); (6) CIMP: Status for the CpG island methylator phenotype (CIMP-H, CIMP-L, CIMP3 and CIMP4); (7) CMS: consensus molecular subtypes (CMS1, CMS2, CMS3 or CMS4). Cell lines that couldn’t be confidently assigned to a single subtype (FDR>;5%) were labeled as NA (not available); (8) CRIS: colorectal cancer intrinsic subtypes (CRIS-A, CRIS-B, CRIS-C, CRIS-D or CRIS-E). Cell lines that couldn’t be confidently assigned to a single subtype (FDR>5%) were labeled as NA (not available); (9-13) APC, TP53, NRAS, KRAS, BRAF: mutational status for these genes, i.e., being wild-type (WT) or mutant (MUT) for each gene. The mutational status was determined based on the presence of somatic mutations that are associated with the alteration of protein sequences (missense SNVs, nonsense SNVs and indels); (14) (15) (16) Aneuploidy score, ploidy, aneuploidy score over ploidy calculated as described in the methods; (17) References: publications in which additional information about each cell line can be found. (c-d). STR (Short Tandem Repeats) profiles of the 88 CRC cell lines, used for the screening and validation dataset, considering 16 different loci.

Additional file 3: Table S2. Characteristics of CRC PDO models. (a) Annotation of the 12 CRC PDOs. For each organoid we report: (1) Sample name; (2) Age of the patients; (3) Site of resection; (4) Primary tumor site; (5) Stage; (6) RAS mutational status; (7) BRAF V600E status; (8) Microsatellite status: microsatellite stable (MSS) or instable (MSI); (9) CIMP: Status for the CpG island methylator phenotype (CIMP-H, CIMP-L, CIMP3 and CIMP4); (b) STR (Short Tandem Repeats) profiles of the 12 PDOs, used for the screening, considering 16 different loci.

Additional file 4: Table S3. Characteristics of CRC PDX models. (a) Annotation of the 490 CRC PDXs. For each sample we report: (1) Sample name; (2) Site of resection; (3) Primary tumor site; (4) Sex; (5) Microsatellite status: microsatellite stable (MSS) or instable (MSI); (6-7) KRAS and BRAF mutational status; (8) Age of the patients; (9) CIMP: Status for the CpG island methylator phenotype (CIMP-H, CIMP-L, CIMP3 and CIMP4).

Additional file 5: Table S4. Quantification of cfDNA release by CRC cell lines. (1) Cell line name; (2) cfDNA value for each cell line; the colors indicate their relative quartiles.

Additional file 6: Table S5. Similarity between intracellular and supernatant DNA based on 13,568,038 genomic positions annotated on hg38 as *common SNPs*.

Additional file 7: Table S6. Quantification of reads from WGS mapping to either chromosomal or mitochondrial DNA in cell line pellets and their corresponding supernatants.

Additional file 8: Table S7. Hallmark annotation of the genes associated with differentially methylated gene promoter regions. The annotation of probes to genes was retrieved from the Infinium HumanMethylation450K manifest file. Genes were then associated with hallmark gene sets from MSigDB collection by using the msigdbr R package.

Additional file 9: Table S8. Methylation data of cfDNA screening dataset. (1) Cell line name; (2) cfDNA screening class: high or low release cfDNA; (3) cfDNA screening quartile; (4) BeadChip microarray used for the methylation analyzes: Infinium Methylation EPIC Kit or HumanMethylation450 BeadChip Kit; (5–146) Probe name and relative values.

Additional file 10: Table S9. Prediction of cfDNA shedding by the methylome classifier in CRC cell lines and PDOs. (1) Cell line name; (2) RPMM cluster: rR high cfDNA release cluster, rL low cfDNA release cluster; (3) Prediction: expected high cfDNA release, expected low cfDNA release; (4) Silhouette width; (5) BeadChip microarray used for the methylation analyzes: Infinium Methylation EPIC Kit or HumanMethylation450 BeadChip Kit; (6–133) Probe name and relative values.

## Data Availability

Raw DNA methylation data (IDAT files) previously obtained for CRC cell lines using the Infinium HumanMethylation450 BeadChip array are available in the Gene Expression Omnibus (GEO) with GSE86078 [14] accession code. Raw DNA methylation data (IDAT files) recently obtained for CRC cell lines using the Infinium MethylationEPIC BeadChip microarray were deposited on GEO with GSE220197 [89] accession code. Raw DNA methylation data (IDAT files) recently obtained for PDOs using the Infinium MethylationEPIC BeadChip microarray were deposited on GEO with GSE274189 (https://www.ncbi.nlm.nih.gov/geo/query/acc.cgi?acc=GSE274189) accession code. The.idat files for all PDX samples are stored at the Gene Expression Omnibus (GEO) with accession number GSE208713 [90]. DNAseq and RNA-seq data previously obtained for CRC cell lines are available in the European Nucleotide Archive (ENA) with PRJEB33045 (https://www.ebi.ac.uk/ena/browser/view/PRJEB33045) [91], PRJEB33640 (https://www.ebi.ac.uk/ena/browser/view/PRJEB33640) [39], and PRJEB57691 (https://www.ebi.ac.uk/ena/browser/view/PRJEB57691) [89] accession codes. Further NGS data (including ATACseq raw data) for CRC cell lines were deposited in ENA with PRJEB78621 (https://www.ebi.ac.uk/ena/browser/view/PRJEB78621) (ERP162918) accession code. All materials and other experimental data are available upon request.

## Abbreviations

CRC: Colorectal cancer
mCRC: Metastatic colorectal cancer
cfDNA: Cell-free DNA
CIMP: CpG Island Methylator Phenotype
CRIS: CRC intrinsic subtypes
CMS: Consensus molecular subtype
MSI: Microsatellite instability
RNA-seq: RNA sequencing
STR: Short Tandem Repeats
WES: Whole-exome sequencing
CIN: Chromosomal instability
WGS: Whole-genome sequencing
