# Supplementary material for: DNA demethylation triggers cell free DNA release in colorectal cancer cells
Source: Genome Med. 2024 Oct 9;16:118. doi: 10.1186/s13073-024-01386-5 (PMC11462661; doi:10.1186/s13073-024-01386-5)

**
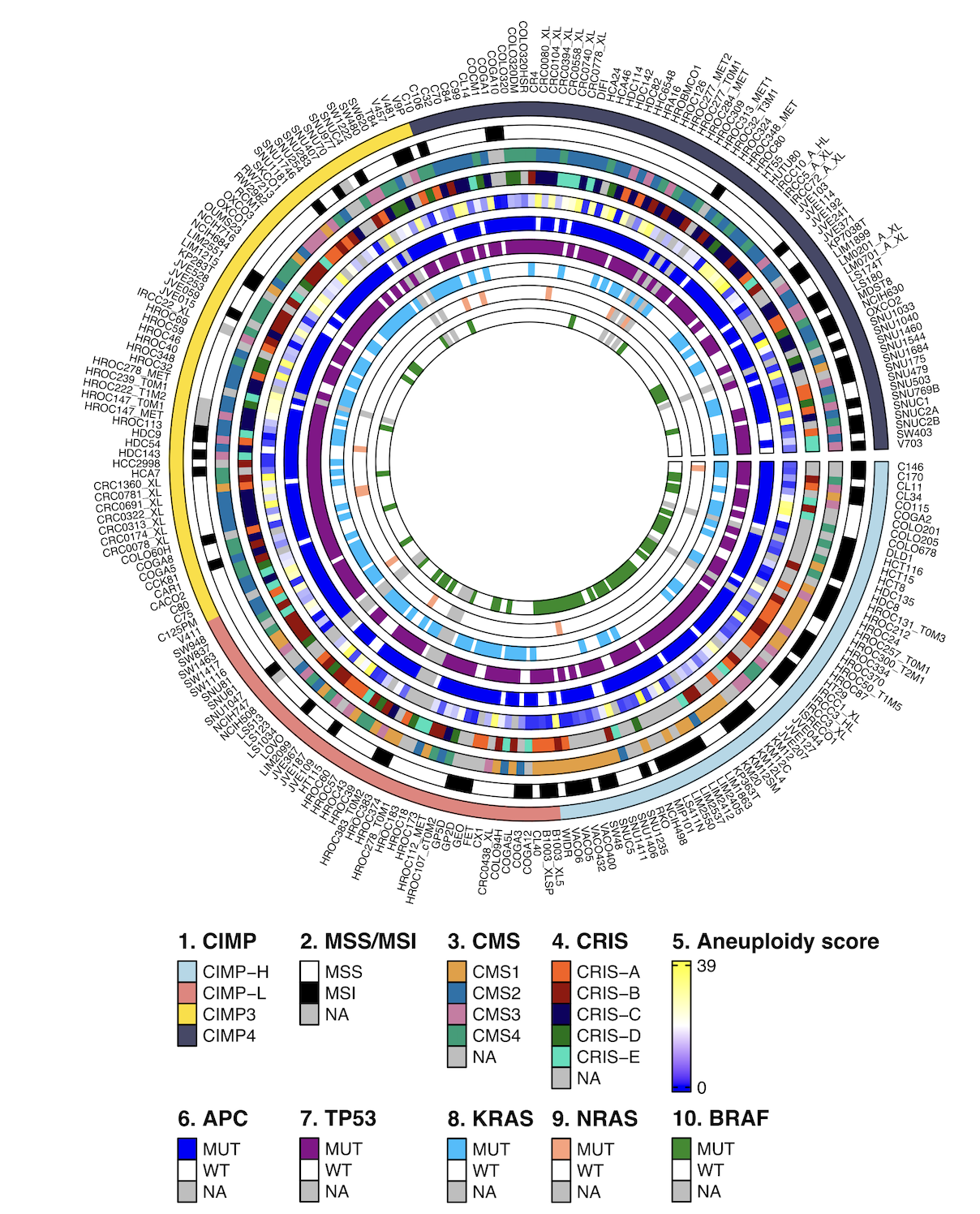
Fig S1. Annotation of 240 CRC cell lines.** Molecular features of the collection of 240 CRC cell lines employed in this study, of which 76 models were used in the screening phase and the remaining 164 were exploited as a validation dataset. From the outside to the inside, the circos plot summarizes the following features: CIMP classification (CIMP-H, CIMP-L, CIMP3 or CIMP4), microsatellite status (MSS or MSI), CMS transcriptional subtypes (CMS1, CMS2, CMS3 or CMS4), CRIS transcriptional subtypes (CRIS-A, CRIS-B, CRIS-C, CRIS-D or CRIS-E), aneuploidy score, APC mutational status, TP53 mutational status, KRAS mutational status, NRAS mutational status, BRAF mutational status. Sample mutational status for the listed genes was determined based on presence of somatic mutations that are associated with the alteration of protein sequences (missense SNVs, nonsense SNVs and indels). In the layers corresponding to CMS and CRIS transcriptional subtypes, cell lines that could not be confidently assigned to a single subtype (FDR>5%) were labelled as NA (not available).**
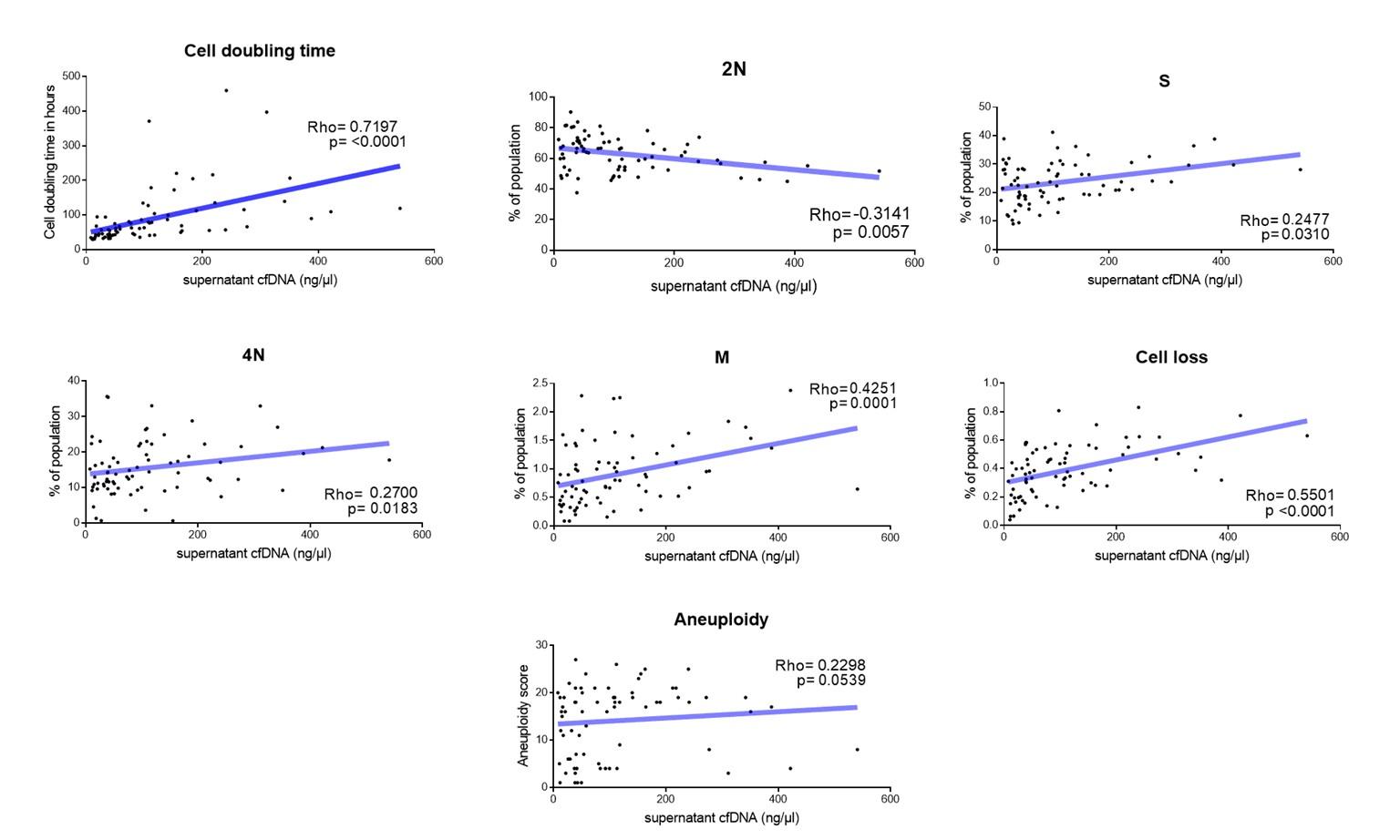
Fig S2. Association between cfDNA values and biological parameters or molecular features.** Correlation analyses between cfDNA amounts with cell doubling time (hours; as determined by Cell Trace Violet incorporation); cell cycle phases (2N, S, 4N, M; as determined by flow cytometry); cell loss index (apoptotic and necrotic cells; assessed by annexin V/ propidium iodide staining) and aneuploidy score based on NGS data. Statistical analyses were performed using Spearman correlation**.Fig S3. Mitotic phases in representative CRC cells from the different cfDNA release quartiles.** The percentage of mitotic cells in the prophase to metaphase stages, compared to anaphase and telophase was observed in the highest cfDNA releasing group.

**Fig S4. Quantification of KRAS mutant allele copies for HCT116 (parental and DKO) in supernatant cfDNA extracted from different volumes of cell supernatants** The number of KRAS mutant allele copies was consistently more abundant in DKO as compared to parental.


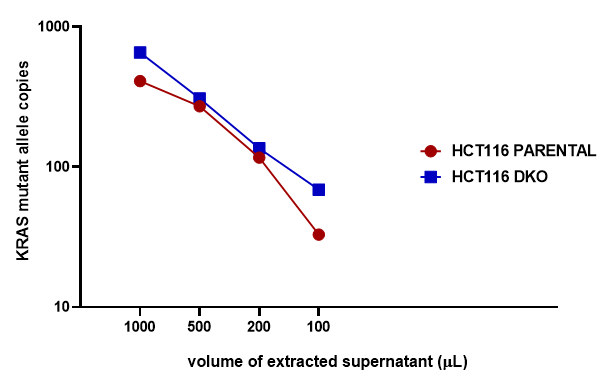


**Fig S5. Pharmacological depletion of DNA methylation stimulates cfDNA release in pancreatic cancer cell lines.** cfDNA shedding was increased in the supernatant of pancreatic cancer cells exposed to nontoxic concentrations of the demethylating agent decitabine. Statistical significance: * P < 0.05.


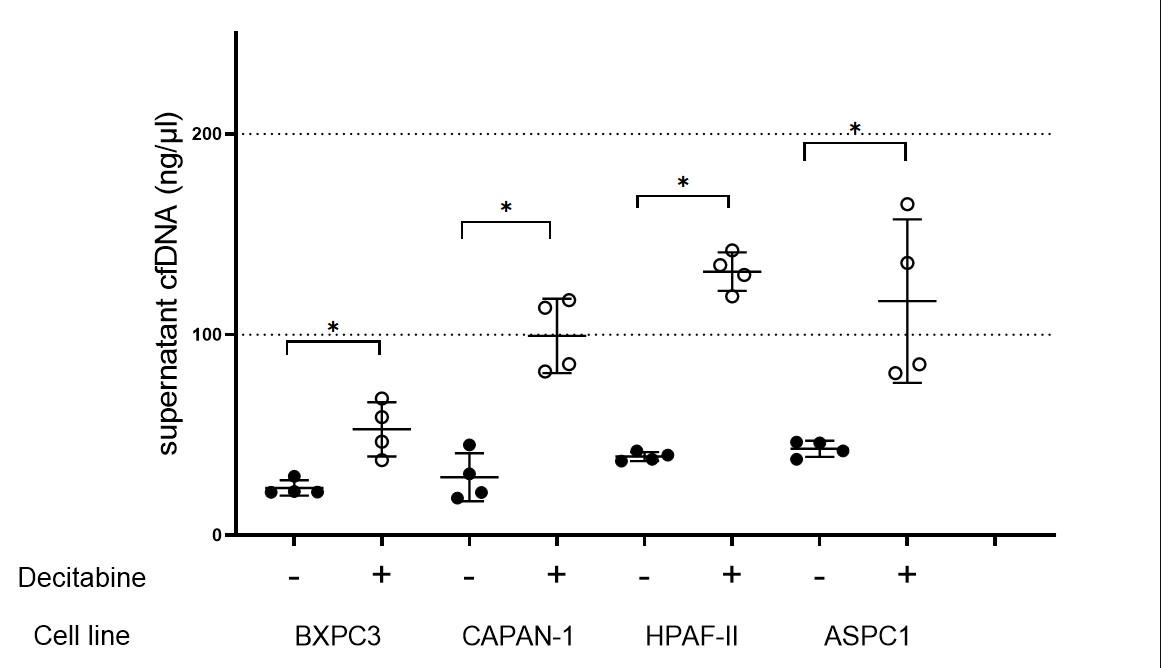

Supplement: Supplementary file 1 — Additional file 1: Fig S1.Annotation of 240 CRC cell lines. Fig S2. Association between cfDNA values and biological parameters or molecular features. Fig S3. Mitotic phases in representative CRC cells from the different cfDNA release quartiles. Fig S4. Quantification of KRAS mutant allele copies for HCT116 (parental and DKO) in supernatant cfDNA extracted from different volumes of cell supernatants. Fig S5. Pharmacological depletion of DNA methylation stimulates cfDNA release in pancreatic cancer cell lines. [file 13073_2024_1386_MOESM1_ESM.docx]
